# Application of TINAVI orthopedic robot-assisted proximal interlocking screw placement for femoral retrograde intramedullary nails: a retrospective clinical study

**DOI:** 10.1007/s11701-025-02787-3

**Published:** 2025-09-13

**Authors:** Yu Su, Zhong Li, Chengcheng Zhang, Xianjie Ai, Bo Wu, Qian Wang, Cheng Ren, Teng Ma, Ming Li

**Affiliations:** 1 Department of Orthopedics and Trauma, Honghui Hospital, Xi’an Jiaotong University, Youyidong Road, Xi’an, 710000 Shaanxi China; 2 Medical College, Yan’an University, Yan’an, Shaanxi China

**Keywords:** TINAVI robot navigation system, Free-hand technology, Retrograde intramedullary nail, Interlocking screw placement, Radiation exposure

## Abstract

Retrograde intramedullary nailing is a common treatment for middle and lower femoral fractures where proximal locking is the main challenge. This study explored the application and experience of robot-assisted retrograde intramedullary nail proximal precision locking. Data from 74 patients with middle and lower femoral fractures treated with retrograde intramedullary nails between January 2021 and June 2023 were retrospectively analyzed. According to the different proximal locking methods, the data were divided into a robotic group (34 cases) and a free-hand group (40 cases). General, perioperative, and follow-up data of the two groups were collected, and their differences were compared based on specified parameters. The results are as follows: the mean follow-up time was (16.10 ± 2.38) months, the number of screw placements in the robotic group (1.03 ± 0.17) was less than that in the manual group (1.63 ± 1.15, *P* = 0.004), and the number of fluoroscopies (1.06 ± 0.34) was significantly less than that in the manual group (6.35 ± 2.46, *P* < 0.001). Additionally, the total length of the proximal incision (2.16 ± 0.24) was shorter than that in the manual group (3.58 ± 0.38, *P* < 0.001), and the success rate of one-time screw placement (97.1%, 33/34) was significantly higher than that in the manual group (67.5%, 27/40; *P* = 0.001). There were no significant differences in the other indices of the two groups (*P* > 0.05). For the treatment of the middle and lower femoral fractures using a femoral retrograde intramedullary nail, the robotic navigation system is more efficient than traditional manual nail placement under fluoroscopy because it can shorten the number of proximal locking nail placements, the number of fluoroscopies, and the total length of the proximal incision. Additionally, it can reduce the exposure to external radiation and increase the success rate of one-time nail placement. Thus, this study presents a novel approach for the placement of proximal interlocking screws in femoral retrograde intramedullary nails, making it worthy of clinical application.

**Supplementary Information:**

The online version contains supplementary material available at 10.1007/s11701-025-02787-3.

## Introduction

Retrograde intramedullary nailing is a widely adopted technique for treating middle and lower femoral fractures. Compared to locking plates, this method offers notable benefits such as central fixation, reduced stress shielding, minimal trauma, fewer complications, and favorable treatment outcomes, as supported by multiple studies [1–6]. However, this technique has some drawbacks, particularly the challenges associated with proximal locking procedures and the risk of nail breakage, with proximal locking constituting the primary obstacle to its application [4, 5, 7–9]. The existing literature indicates that proximal locking accounts for approximately half the overall duration of intramedullary nailing procedures [10]. The conventional approach to proximal locking involves the use of free-hand screw locking guided by fluoroscopy [11]. This method requires continuous intraoperative fluoroscopic imaging to adjust the screw position and angle, thereby facilitating accurate placement. Unfortunately, this process leads to substantial radiation exposure in both the patients and surgical teams [11, 12].

Numerous innovative technologies have been introduced and implemented to ensure precise proximal locking while minimizing radiation risks to both medical personnel and patients. These include remote-targeting mechanical devices [13], endoscope-assisted techniques [8], computer-aided preplanning methods [5], laser navigation [14], and electromagnetic navigation technologies [7, 15]. Each technology has unique advantages and limitations. For instance, the aiming device faces challenges in compensating for positional deformations of up to 18 mm in the lateral position owing to the deformation of the main nail during surgery [16]. The endoscopic technique, which guides nail placement under endoscopic lighting [8], and the computer-aided preplanning method, which predicts the locking nail position after main nail deformation by analyzing preoperative models [5], have demonstrated promising experimental performance. However, their clinical efficacy remains uncertain, and several studies have indicated that laser and electromagnetic navigation techniques outperform free-hand nailing techniques in terms of radiation exposure and accuracy [14]. However, these technologies are associated with high base costs owing to the need for additional equipment and professionals [7, 15]. Furthermore, they have limited applicability in orthopedic surgery, low compatibility, and potential risks of laser and electromagnetic radiation for surgeons [14]. Consequently, no new technology has gained clinical acceptance. Currently, manual locking remains the most prevalent locking method in clinical practice, particularly in major hospitals in most of the regions of China [14–17]. However, the success of this technique relies heavily on the surgeon's proficiency, and the learning curve is relatively long. Given the concerns regarding both the high radiation exposure and screw placement accuracy [7, 8], there is a pressing need to develop new technologies to address these challenges.

In recent years, robotic navigation has gained widespread application in orthopedic surgery primarily because of its high precision and minimal invasiveness. This technology is promising in addressing longstanding challenges in this field [18]. Numerous reports have highlighted the capability of robotic navigation systems to enhance the accuracy and precision of screw locking while significantly reducing the overall radiation exposure [4, 9]. Despite these advancements, there is no clinical report on the study of robot-assisted proximal interlocking screw placement of femoral retrograde intramedullary nail. Therefore, a retrospective analysis of data from 74 patients who met the predefined inclusion and exclusion criteria was conducted in this study. By comparing the robot-assisted proximal interlocking screw placement of retrograde intramedullary nails with the traditional free-hand method, this study aimed to explore its practical application and gain insights into the experience of utilizing robotic assistance for achieving precise proximal locking in femoral retrograde intramedullary nail procedures.

## Materials and methods

### Inclusion and exclusion criteria

Inclusion criteria: according to X-ray and computed tomography (CT); meeting the fracture criteria of middle and lower femoral fractures [19]; age ≥ 18 years; closed fracture; with surgical indications where the patient requires surgery; retrograde intramedullary nail fixation; complete follow-up data.

The exclusion criteria were a poor general condition, inability to tolerate surgery or anesthesia, pathological or old fractures, malignant disease, and refusal to participate in the study.

### General information

This study was a retrospective, clinical, controlled study. From January 2021 to June 2023, 74 patients with middle and lower femoral fractures were treated with retrograde intramedullary nail fixation in Honghui Hospital, which is affiliated with the Medical College of Xi’an Jiaotong University. Based on the proximal locking methods employed, the patients were categorized into two groups: the robotic group (*n* = 34) and the free-hand (or conventional) group (*n* = 40). In the robotic group, proximal locking was performed using the TINAVI orthopedic robot navigation system, whereas in the free-hand group, the traditional free-hand nail placement technique was used to accomplish proximal locking.

**Fig. 1:**
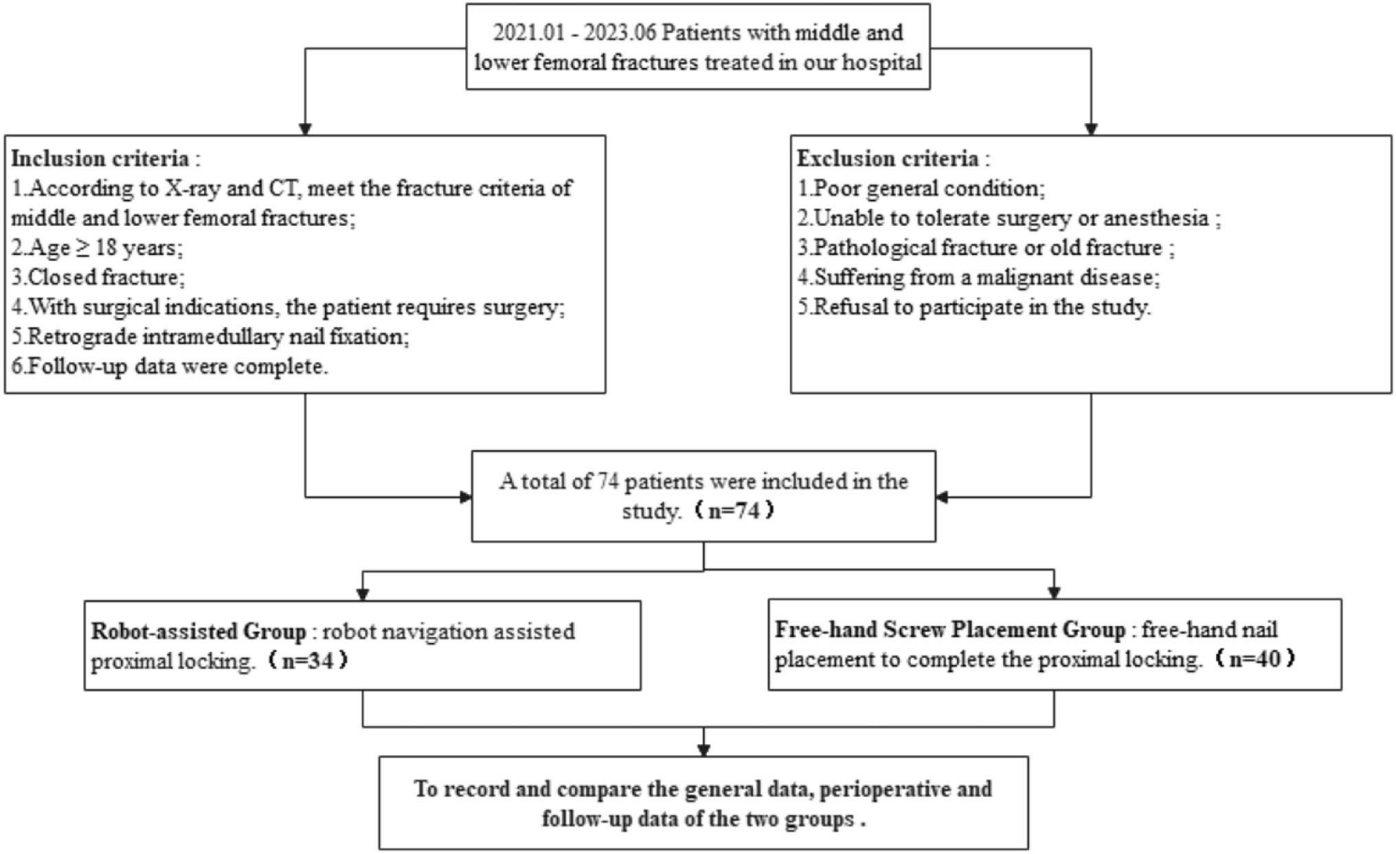
Flowchart of the process

This study was approved by the Ethics Committee of Honghui Hospital, Xi’an Jiaotong University (approval number: 202410010). All findings were reported in accordance with the Strobe Statement guidelines, and the study was conducted in accordance with the Declaration of Helsinki. Informed consent was obtained from all the participants and/or their legal guardians. A flowchart depicting the research process is provided in Fig. 1.

### Research methods

#### Preoperative preparation

All patients were admitted to the hospital for comprehensive diagnostic evaluation. Radiographic and CT were performed to assess the fracture site, and symptomatic treatments, including the management of swelling, immobilization, and prophylaxis against deep venous thrombosis of the lower extremities, were administered. In cases where patients presented with severe comorbidities, consultations with relevant specialist departments and the anesthesiology department were sought to evaluate the patient’s surgical tolerability.

#### Surgical methods

The surgical procedure was performed under general anesthesia combined with a nerve block, with the patient in the supine position, and following routine sterilization and draping protocols. The affected limb was positioned neutrally in the lumbosacral region, and a sterile roll was placed behind the knee to maintain 30° of flexion, facilitating the ease of repositioning and stabilization. A medial parapatellar incision of approximately 4 cm in length was made, and the layers were exposed sequentially. The medial border of the patellar tendon was used to access the joint cavity, allowing the full visualization of the femoral intercondylar fossa. The insertion of the posterior cruciate ligament was then identified as the entry point. Under lateral fluoroscopic guidance, this point was aligned with the apex of the “V” formed by the anterior femoral condyle and the Blumensaat line. Subsequently, a guide needle was inserted into the proximal femoral medullary cavity, and closed reduction was achieved under longitudinal traction. In instances where reduction was challenging, a 1-cm longitudinal incision was made to aid in the process. Once the femoral length was restored, the positions of the guide pin and fracture ends were verified using C-arm fluoroscopy. The main nail (supplied by Tianjin Zhengtian Industrial Co., Ltd.) was then inserted incrementally and guided by a needle, and its length and position were confirmed via fluoroscopy. The distal screw was placed using an aiming device to secure the distal femur, followed by a proximal locking procedure.

For the robot-assisted group, the TINAVI orthopedic robot (manufactured by Beijing Tianzhihang Medical Technology Co., Ltd.) was installed. The tracers were positioned on the distal femur facing the foot. Fluoroscopic images were then obtained using a C-arm machine, and anteroposterior and lateral radiographs of the femur were imported into the robotic system. The system was used to plan the placement of the proximal locking nail on the femoral retrograde intramedullary nail. After confirming the optimal circular trajectory, the operator, assisted by robotic navigation, placed a proximal screw on the contralateral side to stabilize the proximal femur.

In the free-hand screw placement group, the ideal circular trajectory was identified using repeated fluoroscopic imaging. The proximal screw was then placed under direct visual guidance with repeated fluoroscopic verification to ensure precise screw positioning.

**Fig. 2:**
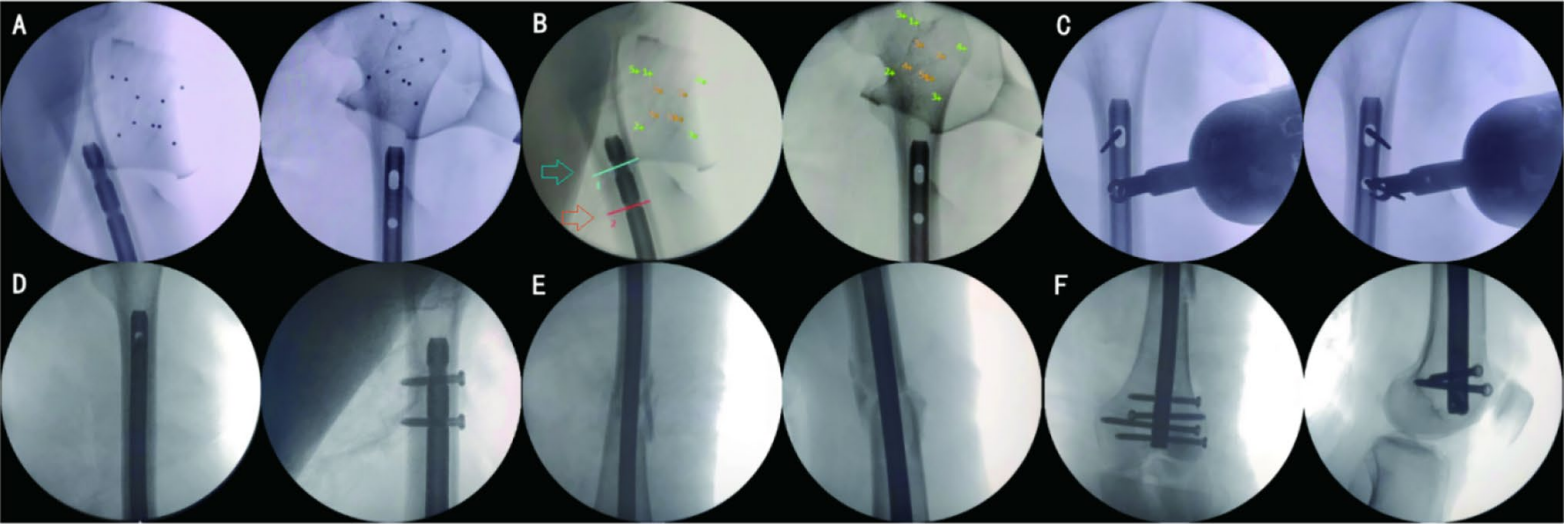
Robot-assisted group proximal locking. **A** Anteroposterior and lateral X-ray films of the proximal femur; **B** planning of the two proximal locking screw placement routes (green and red arrows) on the robot navigation system; and **C** Kirschner wire placed along the guide, and its confirmed position via fluoroscopy. **D** Precise placement of the proximal locking screw; **E** fracture end; and **F** distal screw placement

Upon completion of proximal locking, an appropriately sized tail cap was secured at the distal end. The final confirmation of satisfactory fracture reduction, as well as the correct positioning of the intramedullary nail and screw length, was achieved through fluoroscopy. The instruments were then accurately accounted for, a drainage tube was placed, and the wound was thoroughly irrigated and sutured (Fig. 2).

In this study, all operations in the robotic and free-hand groups were completed by the same group of senior surgeons. The surgeon had rich surgical experience with free-hand screw placement and robot-assisted screw placement.

#### Postoperative treatment

Both groups received identical postoperative care, which included the routine administration of antibiotics to prevent infection, use of an analgesic pump for pain management, and dressing Changes within the first 24 h postoperatively. The decision to remove the drainage tube was based on the drainage status of each patient. Postoperative imaging examinations were conducted as part of the follow-up protocol, and the patients were encouraged to perform isometric contractions of the affected limb muscle groups during the early postoperative period, followed by a gradual exercise program to restore joint function. Regular postoperative reviews were then scheduled, and the patients were gradually transitioned to full weight bearing as callus formation progressed.

#### Observation indicators and evaluation criteria

In both groups, several parameters were recorded, including the number of screw placements, fluoroscopy times, success rate of one-time screw placement, screw placement duration (defined as the time from drilling to successful locking), total length of the proximal incision, and intraoperative blood loss. In addition, the overall operative time, measured from the initial wound incision until the completion of suturing, was documented. Postoperative follow-up was then performed in an outpatient setting. During the follow-up period, the patient’s complications were recorded, and bone healing was observed using radiography. The fracture healing standard was based on the presence of a continuous callus line through the fracture line on the X-ray image and the complete disappearance of the fracture line. At the 12-month postoperative mark, the Hospital for Special Surgery (HSS) knee function score was used to evaluate knee function.

#### Statistical methods

Data analyses were performed using the Statistical Package for the Social Sciences (SPSS for Windows, version 18.0, Chicago, SPSS Inc.). The dataset included general, perioperative, and follow-up data. Specifically, the perioperative and follow-up data included the number of screw placements, number of fluoroscopies performed, success rate of one-time screw placement, duration of screw placement, and total length of the proximal incision. Additionally, it also included the intraoperative blood loss, overall operation time, time to fracture healing, HSS knee function score one year after the operation, and complications and reoperation rate. Measurement data were presented as the mean ± standard deviation (SD), and were subjected to a normality test followed by a two-sample independent t-test for analysis. The count data were expressed as the number of cases and percentages (%), and were analyzed using the chi-square test or Fisher’s exact probability method. A statistical significance level of 5% (*P* < 0.05) was set for all analyses.

## Results

### General information

No significant differences were observed between the two groups in terms of age, sex, body mass index (BMI), side of injury, cause of injury, Arbeitsgemeinschaft für Osteosynthesefragen/Orthopedic Trauma Association (AO/OTA) fracture classification, and the time interval from injury to operation (all P > 0.05), indicating comparability between the groups (Table 1). All patients achieved clinical healing without any serious complications such as deep infections, internal fixation failures, or the need for secondary surgeries. The mean follow-up duration was 16.10 ± 2.38 months (range: 12–21 months), and no significant differences in the follow-up time were detected between the two groups (*P* = 0.873) (Table 2).

**Table 1:** Comparison of preoperative general data

Factor	Robotic group(*n* = 34)	Free-hand group (*n* = 40)	*χ*^2^/t value	*P*-value
Age, (years)	47.3 ± 15.15	45.3 ± 14.48	0.601	0.550
Gender, *n* (%)			0.064	0.800
Male	22(64.7%)	27(67.5%)		
Famale	12(35.3%)	13(32.5%)		
BMI	22.66 ± 4.97	23.16 ± 5.07	0.431	0.668
Injured side, *n* (%)			0.001	0.970
Left	16(47.1%)	21(52.5%)		
Right	18(52.9%)	19(47.5%)		
Injury causes, *n* (%)			0.035	0.982
Traffic trauma	8(23.5%)	10(22.5%)		
Fall injury	23(67.6%)	28(67.5%)		
High falling injury	3(8.8%)	4(10%)		
AO/OTA fracture classification, *n* (%)			0.067	0.967
A	28(82.4%)	33(82.5%)		
B	3(8.8%)	4(10.0%)		
C	3(8.8%)	3(7.5%)		
Operation time caused by injury (days)	4.59 ± 2.54	4.78 ± 2.63	0.310	0.758

**Table 2:** Comparison of intraoperative and postoperative data

Factor	Robotic group (n = 34)	Free-hand group(n = 40)	*χ*^2^/t value	*P*-value
Number of nail placements (times)	**1.03 ± 0.17**	**1.63 ± 1.15**	**2.295**	**0.004**
Screw perspective times (times)	**1.06 ± 0.34**	**6.35 ± 2.46**	**12.451**	** < 0.001**
One-time successful screw placement rate (%)	**97.1%(33/34)**	**67.5%(27/40)**	**10.468**	**0.001**
Nail placement time (min)	9.49 ± 1.86	10.34 ± 5.47	0.866	0.390
Total operation time (min)	198.27 ± 58.77	210.28 ± 61.83	0.852	0.397
Total length of proximal incision (cm)	**2.16 ± 0.24**	**3.58 ± 0.38**	**18.915**	** < 0.001**
Intraoperative blood loss (ml)	115.65 ± 10.53	120.99 ± 11.29	0.827	0.657
Follow-up time (months)	16.05 ± 2.37	16.14 ± 2.41	0.161	0.873
Fracture healing time (months)	5.66 ± 1.29	5.81 ± 1.32	0.479	0.634
Knee joint HSS function score	84.12 ± 2.79	83.38 ± 2.71	1.161	0.250
Complications rate, *n* (%)	2 (5.9%)	4 (10%)	0.544	0.678
Chronic pain	2	3		
Delayed healing	0	1		
Reoperation rate, *n* (%)	2 (5.8%)	3 (7.5%)	0.747	1.000

Bold font indicates that the results are statistically significant

### Perioperative data

In the robotic group, the mean number of screw placements for proximal locking was 1.03 ± 0.17, compared to 1.63 ± 1.15 in the free-hand group. The average number of fluoroscopies was 1.06 ± 0.34 in the robotic group and 6.35 ± 2.46 in the free-hand group. However, the success rate of one-time screw placement was significantly higher in the robotic group (97.1%, 33/34) than in the free-hand group (67.5%, 27/40). Additionally, the total length of the proximal incision was shorter in the robotic group (2.16 ± 0.24 cm) compared to the free-hand group (3.58 ± 0.38 cm). Statistically significant differences in the number of proximal locking screws (*P* = 0.004), number of fluoroscopies (*P* < 0.001), success rate of one-time screw placement (*P* = 0.001), and total length of proximal incision (*P* < 0.001) were also found between the two groups (Table 2).

In the robotic group, the average time for proximal locking was 9.49 ± 1.86 min, whereas that in the free-hand group was 10.34 ± 5.47 min. The mean total operation time was 198.27 ± 58.77 min for the robotic group and 210.28 ± 61.83 min for the free-hand group. Additionally, the intraoperative blood loss was 115.65 ± 10.53 ml in the robotic group and 120.99 ± 11.29 ml in the free-hand group. However, there were no significant differences in the screw placement time (*P* = 0.390), total operation time (*P* = 0.397), and intraoperative blood loss (*P* = 0.657) between the two groups (both *P* > 0.05) (Table 2).

### Follow-up data

In the robotic group, the mean fracture healing time was 5.66 ± 1.29 months, while in the free-hand group, it was 5.81 ± 1.32 months. At the final follow-up, the average HSS score was 84.12 ± 2.79 in the robotic group and 83.38 ± 2.71 in the free-hand group. No significant differences were detected between the two groups regarding fracture healing time (*P* = 0.634) and knee function score (*P* = 0.250) (both *P* > 0.05) **(**Table 2**)**.

There were two cases of complications in the robot group, all of which were chronic pain of knee joint after fracture surgery (5.8%, 2/34). There were four cases of complications in the free-hand group, three3 cases of chronic pain of knee joint, and one case of delayed healing (10%, 4/40). Among them, five patients with chronic pain underwent selective internal fixation removal after fracture healing, and the above pain symptoms were significantly relieved after operation. The reoperation rates of the two groups were 5.9% (2/34) and 7.5% (3/40), respectively. Patients with delayed healing were found to have fracture healing at 10 months after surgery by quitting smoking and strengthening nutrition. There were no significant differences in the incidence of complications (*P* = 0.544) and reoperation rate (*P* = 0.747) between the two groups (both *P* > 0.05) (Table 2).

## Discussion

Fractures of the middle and lower femurs, which are located adjacent to the knee joint, are characterized by extensive muscle and soft tissue attachments, often resulting in the significant displacement of the fracture ends owing to muscle traction. Inadequate management following injury can lead to joint deformity and limited mobility, severely affecting the patients ’ quality of life. Consequently, surgical intervention is recommended for these fractures. Both retrograde intramedullary nails and locking plates are viable options for fracture fixation [1, 20, 21]. Compared with locking plates, retrograde intramedullary nails offer notable biomechanical advantages through indirect reduction and central fixation. Furthermore, they minimize the damage to the periosteum, soft tissues, and blood supply, resulting in lower rates of fracture infection and non-union. As such, this method has gained widespread acceptance for the treatment of middle and lower femoral fractures [22–24]. However, the insertion of the intramedullary nail’s main component can be challenging owing to the elasticity of the metal and the irregularity of the long bone marrow cavity, potentially leading to the failure of the manufacturer-provided external aiming device, particularly during proximal locking [16]. Traditional manual locking under fluoroscopy necessitates repeated drilling and imaging, which increases radiation exposure and may cause additional bone and soft tissue trauma [24, 25]. Failure of proximal locking can result in iatrogenic fractures and the instability of internal fixation, potentially leading to severe limb dislocation. Thus, proximal locking remains a significant challenge in this technique [5]. In recent years, domestic TINAVI orthopedic robot navigation systems have been increasingly used in orthopedic surgery, and their precise positioning capabilities hold promise for addressing this issue [9, 26].

Robot-assisted navigation systems have gained widespread application in orthopedic surgeries, including spine, joint, and trauma procedures, with favorable clinical outcomes [18, 27–31]. Numerous studies have substantiated that robot-assisted navigation offers superior accuracy and safety compared to manual techniques, aligning with the principles of minimally invasive orthopedic surgery. The interlocking fixation of femoral intramedullary nails remains a challenging aspect for numerous clinicians, particularly those in primary practice. It has been reported by scholars that robot navigation can be used to assist interlocking fixation of intramedullary nails through cadaver detection experiments [9]. Previous studies have focused on the use of robot navigation systems for the positioning and placement of intramedullary nail openings, and there has been no relevant clinical study on the robot for intramedullary nail locking nail placement. In this study, we aimed to assess the placement of proximal interlocking screws for retrograde intramedullary nails using the TINAVI orthopedic robot navigation system. We compared this approach with the conventional free-hand method to investigate the application and experience of the robot-assisted precise proximal locking of retrograde intramedullary nails.

In this study, we compared the perioperative and postoperative follow-up results of the two groups of patients. The results showed that the number of nail placements and fluoroscopy in the proximal locking of the robotic group was lower, and the one-time success rate was higher (*P* < 0.05). The success rate of one-time screw placement in 33 patients in the robotic group was 97.1%, and most patients only needed one fluoroscopy to confirm whether the screw position was satisfactory. If an unarmed screw placement is performed, multiple screw placements may occur owing to various reasons, even if the surgeon is a senior physician with extensive surgical experience. Additionally, repeated fluoroscopy is required to confirm the screw position during this process, which increases the risk of exposure of patients and surgical teams to radiation and related cancers [32, 33]. A study on the distal locking of tibial intramedullary nails reported that the radiation exposure time of distal locking using the navigation system was much shorter than that of the free-hand group (2.13 ± 0.73 s vs 19.09 ± 10.41 s, *P* < 0.05) [12], which was similar to our results. Robotic navigation assists surgeons in achieving accurate and safe proximal locking, mitigating related risks, and enhancing the patient's prognosis. Notably, there were no significant differences in the proximal locking screw placement time and total operation time between the two groups (*P* > 0.05). However, the robotic group demonstrated a relatively stable proximal locking time with a longer planning phase but a shorter operation time, eliminating the need for repeated fluoroscopies during nailing and reducing radiation exposure. Conversely, the free-hand group exhibited a longer nail placement operation time that was highly dependent on the surgeon's experience and posed challenges for novice physicians. In the future, owing to the progress of science and technology, the operation time is expected to be reduced by enhancing the speed of robot planning and navigation. Additionally, free-hand placement may lead to repeated drilling, resulting in additional soft tissue and bone trauma, along with a longer total length of the proximal incision and more pronounced postoperative pain in patients [4]. Finally, there were no significant differences in the fracture healing time, knee function score at 12 months after surgery, and complication and reoperation rates between the two groups (*P* > 0.05), indicating that the use of a robotic navigation system for the proximal locking of retrograde intramedullary nails in the treatment of middle and lower femoral fractures did not affect the recovery of knee function. Five patients in the study had chronic knee pain, which was considered to be related to the placement of the retrograde intramedullary nails and surgical approaches. Although the symptoms were significantly reduced after internal fixation removal, the pressure of patient life during the treatment cycle and the economic pressure caused by secondary surgery are issues that clinicians cannot ignore. Proximal locking assisted by robotic navigation can decrease the number of screw placements and fluoroscopies, reduce external radiation exposure, shorten the total length of the proximal incision, and leverage the advantages of robotic precision and minimally invasive techniques.

However, mastering robotic navigation surgery incurs a long training time; therefore, the operation time and research conclusions may depend on the surgeons’ stages of the learning curve. Some scholars have studied the learning curve of pedicle screw implantation under robot navigation and found that the average scanning time decreased from (15.4 ± 7.8) min to (8.4 ± 3.3) min (comparison of the first phase with the fourth phase), and the accuracy of screw placement increased from the initial 83.1% to 95.1% (*P* < 0.001) [34], indicating that different learning curves affect the research conclusions. Considering these limitations, the surgeries in this study were performed by the same group of senior surgeons, who have extensive surgical experience and can skillfully master robot navigation systems. Therefore, the learning curve has little effect on the results of this study.

Nousiainen et al. postulated that the advent of novel technologies not only presents new avenues for therapeutic interventions but also evolves into a potent instructional instrument for imparting surgical skills [35]. Building on this hypothesis, several researchers have conducted comparative analyses between conventional fluoroscopy and electromagnetic navigation guidance to examine their impact on the execution of the distal locking of the tibial intramedullary nails by novice practitioners. Their findings revealed that new technology enhances operational efficiency and precision, mitigates radiation exposure, and augments the novices’ motivation to learn, while abrogating the timeframe for acquiring new competencies [12]. Our results corroborate these observations. Specifically, we identified that the proximal locking of femoral retrograde intramedullary nails poses an additional challenge. Historically, this procedure has necessitated the collaboration of seasoned physicians with specialized C-arm technicians, thereby increasing operational prerequisites [8]. Furthermore, an extended learning curve and substantial radiation exposure tend to dampen the enthusiasm of novice doctors [14]. During our study, we observed a preference among junior doctors in the experimental group for robotic navigation assistance over manual screw insertion. The integration of innovative robotic navigation technology not only diminishes radiation exposure but also alleviates surgical complexity, enabling even inexperienced practitioners to successfully perform the procedure with robotic aid. Moreover, the introduction of these innovative auxiliary devices reduces foundational prerequisites and complexity of surgeries, stimulates the learning interest of young doctors, shortens the learning curve associated with intricate surgical techniques, and fosters the development of primary care physicians. It should be noted that although the application of the robot navigation system is beneficial to doctors and patients, it has not been widely used in clinical practice due to its high cost of purchase, maintenance, and use. However, it is believed that with the development of the times, especially the continuous progress of AI artificial intelligence technology, robots will be widely used and beneficial to human life in the future.

However, despite successful positioning, a discrepancy in the screw placement direction might still have occurred during our investigation [8, 36]. This phenomenon can be attributed to the screw insertion point being located on a slope where the guide needle is prone to slipping during insertion, ultimately resulting in directional deviations [37, 38]. To address these issues, we adopted the following surgical technique: upon verifying that the guide and nail hole were concentric, a 2.5 mm diameter Kirschner wire was employed to penetrate a single layer of cortex along the guide. It was crucial to ensure that the drill remained parallel to the guide throughout this process. After drilling, the Kirschner wire was gently brought into contact with the bone surface before proceeding to the anchor and continuing drilling. Under fluoroscopic guidance, the correct position was confirmed, and a hollow drill was used to penetrate the guide, ultimately ensuring precise screw locking. Furthermore, surgeons must be mindful of the layout of the operating rooms. Given that the C-arm machine and TINAVI orthopedic machine required for fluoroscopy are critical orthopedic equipment, an improper layout can directly affect operation duration and fluidity [39]. Based on our experience, we recommend positioning the robot arm and optical tracking system on the affected side, the intelligent navigation platform at the foot end, and the C-arm machine on the opposite side. This configuration provides the surgeon with ample operating space to effectively complete the proximal screw-locking procedure.

This study had some limitations. First, this was a controlled single-center retrospective clinical study with a small sample size and a short follow-up time. Although there was a control group, the lack of randomization in retrospective studies increases the risk of data bias. Second, the high purchase, maintenance, and use costs of robotic navigation affect its clinical applicability; therefore, it cannot be widely performed in the clinic in the short term, especially at the grassroots level. In addition, although the difference in the number of fluoroscopies can indirectly reflect the degree of radiation exposure experienced by physicians to some extent, this study did not accurately quantify and compare the radiation exposure doses. Future studies should consider cooperating with medical centers in other regions to expand the sample size, quantify radiation exposure, and establish multicenter, prospective, and randomized controlled studies.

## Conclusions

In the treatment of middle and lower femoral fractures using femoral retrograde intramedullary nails, the robotic navigation system is more advantageous than traditional manual nail placement under fluoroscopy because it can reduce the number of proximal locking nails and fluoroscopies, the total length of the proximal incision, and the exposure to in vitro radiation. Additionally, it can also increase the success rate of one-time nail placement. This provides a new concept for the placement of proximal interlocking screws in femoral retrograde intramedullary nails, making it worthy of clinical application.

## Supplementary Information

Below is the link to the electronic supplementary material.

Supplementary file1 (PNG 211 KB)

Supplementary file2 (PNG 860 KB)

## Data Availability

The datasets generated and/or analyzed in the current study are available from the corresponding author upon reasonable request.

## Abbreviations

AO: Arbeitsgemeinschaft für Osteosynthesefragen
OTA: Orthopedic Trauma Association
BMI: Body mass index
CT: Computed tomography
HSS: Hospital for Special Surgery
SD: Standard deviation
