# Supplementary figures and images for: Application of TINAVI orthopedic robot-assisted proximal interlocking screw placement for femoral retrograde intramedullary nails: a retrospective clinical study
Source: J Robot Surg. 2025 Sep 13;19(1):599. doi: 10.1007/s11701-025-02787-3 (PMC12433442; doi:10.1007/s11701-025-02787-3)

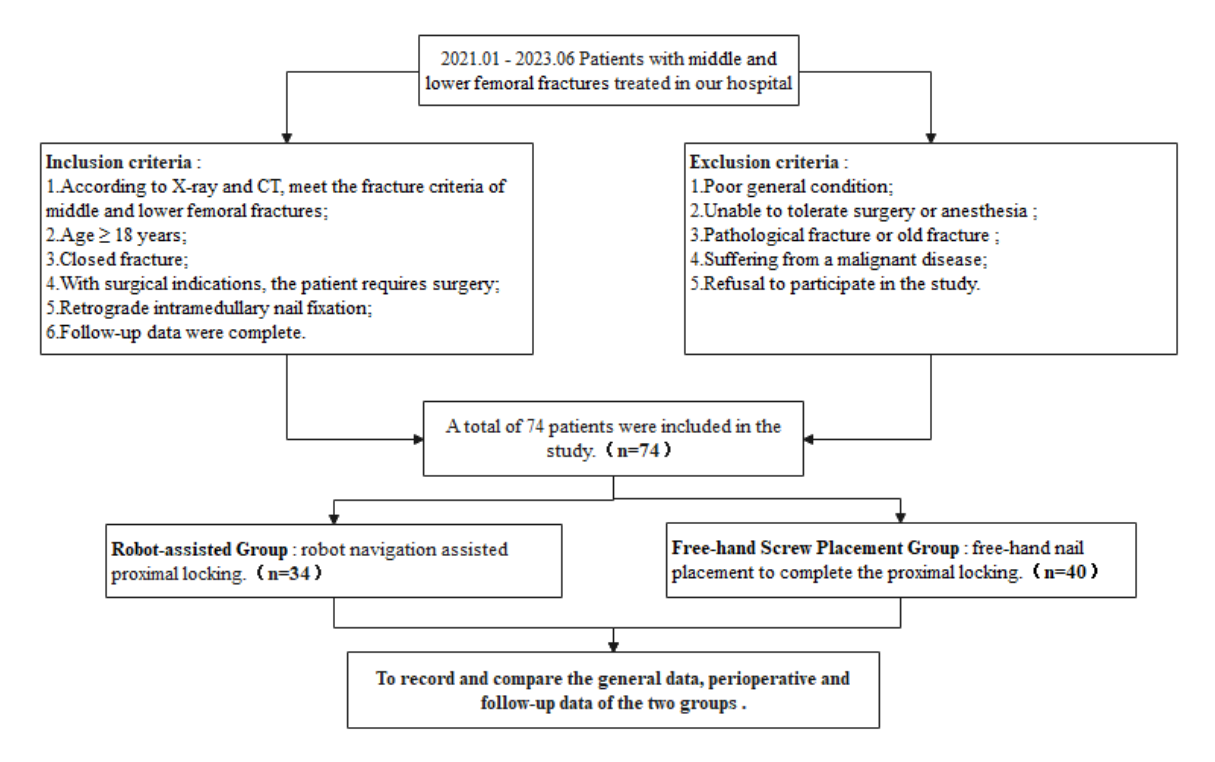

Supplement: Supplementary file 1 — Supplementary file1 (PNG 211 KB) [file 11701_2025_2787_MOESM1_ESM.png]

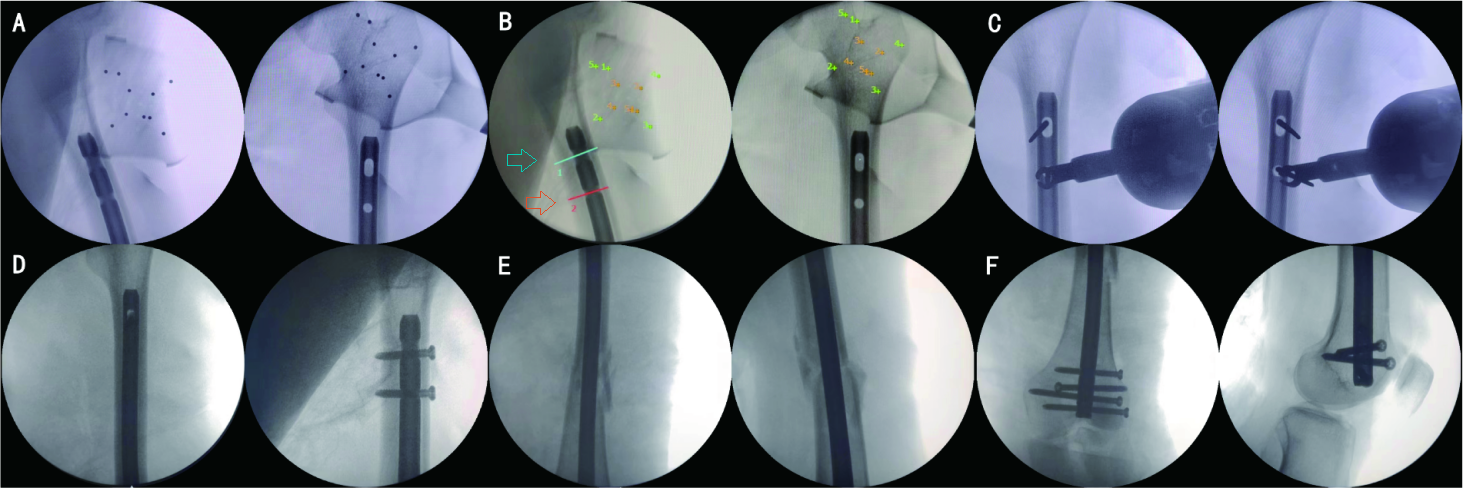

Supplement: Supplementary file 2 — Supplementary file2 (PNG 860 KB) [file 11701_2025_2787_MOESM2_ESM.png]
